# Editorial: Immunoglobulin Glycosylation Analysis: State-of-the-Art Methods and Applications in Immunology

**DOI:** 10.3389/fimmu.2022.923393

**Published:** 2022-05-20

**Authors:** Irena Trbojević-Akmačić, Mohamed Abdel-Mohsen, David Falck, Erdmann Rapp

**Affiliations:** ^1^ Glycoscience Research Laboratory, Genos Ltd, Zagreb, Croatia; ^2^ The Wistar Institute, Philadelphia, PA, United States; ^3^ Leiden University Medical Center, Center for Proteomics and Metabolomics, Leiden, Netherlands; ^4^ Max Planck Institute for Dynamics of Complex Technical Systems, Magdeburg, Germany; ^5^ glyXera GmbH, Magdeburg, Germany

**Keywords:** Fc receptors, glycosylation, glycomics, IgG, immunoglobulins, inflammation, infection

Immunoglobulins (Igs) are critical in the ability of our immune system to recognize and eliminate pathogenic antigens. Igs are co- and post-translationally modified by oligosaccharides (glycans) that significantly impact their structural and functional properties. All five human Ig classes (IgG, IgA, IgM, IgD, and IgE) are glycosylated; and the glycosylation of IgG, in particular, has been studied in depth. Although IgG glycosylation varies between individuals, it is stable within an individual under homeostasis. During disease, IgG glycans can dramatically change making them promising early diagnostic and prognostic biomarkers for several inflammatory, infectious, and autoimmune conditions. The goal of this Research Topic is to cover 1) advancements in analytical approaches for Ig glycosylation analysis; 2) applications of state-of-the-art methodology/technology in exploring aberrant glycosylation patterns during illness/disease; and 3) recent findings on the functional relevance of Ig glycosylation in immunity during different physiological states.

Development of new analytical approaches facilitates the expansion of our knowledge of Ig glycosylation – from its regulation and functional effects in healthy conditions to changes in glycosylation before/during disease, their connection with disease progression, and success of therapeutic interventions. Sensitivity, simplicity, and throughput are three key aims of current methodological development. The GlycoFibroTyper platform of Scott et al. exemplifies this quest for robust methods using minute amounts of sample to tackle large numbers in patients and/or longitudinal samples. The authors combine an antibody capture slide array with direct detection by matrix-assisted laser desorption/ionization (MALDI) (imaging) mass spectrometry (IMS) of PNGase F-released N-glycans – both microarrays and MALDI-MS are well established in high-throughput glycomics. The minimal sample preparation deviates from the dominant liquid chromatography (LC)-MS or LC-fluorescence approaches (1). Though much method development is still focused on IgG, the current advances in sensitivity will hopefully make the analysis of the remaining Ig isotypes more commonplace in the future (2).

IgG glycosylation depends on several demographic, genetic, and environmental factors (such as age, sex, ethnicity, and exercise). Genetic studies provide information on glycosylation regulation. We still know little due to the lack of a direct genetic template and the complexity of glycosylation. Li et al. aim at filling this gap by providing an atlas of genetic regulatory loci related to IgG N-glycosylation with their target genes within functionally relevant tissues. This work implies that the IgG N-glycome is specific for individual tissues. Thereby, it consistently goes beyond general genome-wide association studies (GWAS) (3) in recognizing the impact of the B cell microenvironment (4). The latter also seems (indirectly) affected by estrogen. For example, Mijakovac et al. have explored estrogens impact on IgG glycosylation in the context of menopausal changes.

Glycosylation of Igs is shown to change in various pathological states and has been studied in different diseases. Nevertheless, mechanisms of these changes are still largely unexplored. The main question whether glycosylation changes are a cause or consequence (or both) of disease mostly remains unanswered. IgG glycosylation has been recently studied in the context of thyroid autoimmunity, Hashimoto’s thyroiditis and Graves’ disease, by Trzos et al., connecting IgG glycosylation and Hashimoto’s thyroiditis severity as well as demonstrating an impact of immunosuppressive methimazole therapy on the IgG N-glycome in Graves’ disease. A further study in the parasitic disease lymphatic filariasis by Adjobimey and Hoerauf demonstrates the broad relevance of the IgG N-glycome. However, this fuels the need to control for common comorbidities, for example with endemic controls. Distinct glycan profiles have been observed in endemic normal, asymptomatic individuals bearing microfilaria and patients with chronic pathology. Most notably, agalactosylated and afucosylated IgG distinguished chronic from asymptomatic patients. Linking immune competence and IgG N-glycome composition, detailed characterization on the level of individual subclasses, or antigen-specific antibodies would provide even more insight into these diseases and underlying mechanisms. The IgG subclass-specific glycosylation signatures of liver fibrosis stages obtained by Scott et al. may serve as an example. Demonstrating a greater diagnostic performance than other non-invasive liver fibrosis tests, e.g. APRI, FIB4, their comparably simple workflow highlights the potential attractiveness of IgG glycome analysis for future clinical practice.

The functional relevance of IgG glycosylation is well established and current knowledge of IgG glycosylation in different physiological states is reviewed by Zlatina and Galuska. One of the most established examples of functional effects of glycosylation is core fucosylation at Asn297 of the IgG Fc fragment that results in lower affinity toward Fcγ receptor (FcγR) IIIa, decreasing antibody-dependent cell-mediated cytotoxicity (ADCC)(Sun et al.). Various functions and pathways of Ig sialylation, another important regulator of Ig effector functions, have been highlighted by Vattepu et al. Consequently, glycoengineering has been developed to improve the anti-inflammatory properties of intravenous immunoglobulin (IVIg) often used as a treatment for different inflammatory and autoimmune diseases (Vattepu et al.). IVIg likely has many mechanisms of action although it’s often reduced to sialylated IgG glycans that diminish the affinity of IgG for activating FcγRs and promote recognition by DC-SIGN leading to the expression of inhibitory FcγRIIb. Research by Mimura et al. demonstrates that galactosylated nonfucosylated IgG, which has a high affinity for FcγRIIIa, has a 20 times higher potency to inhibit ADCC compared to native IgG. These findings indicate that IVIg anti-inflammatory activity is partially mediated by blocking FcγRs on immune cells preventing activation, for example by autoantibodies. Glycoengineered recombinant Fc proteins may be a more efficient alternative to plasma-derived IVIg in inflammatory conditions, although binding to the low-affinity FcγRs is not entirely independent of the antigen-binding fragment. Effects of antibody glycoengineering could be tested on e.g. rhesus macaques, a common non-human primate model. The work of Crowley et al. expands our knowledge on the preferences of macaque FcγRs to specific human IgG1 glycovariants and demonstrates this model species’ suitability for evaluation of FcγRs affinity glycoforms. While Ig(G) glycosylation and its functionality are mostly studied in humans and mice, analogous studies in other animals are still lacking, leaving this area largely unexplored. Current knowledge on Ig glycosylation in farm animals is reviewed by Zlatina and Galuska revealing that there are notable differences in Ig glycosylation between animals.

## Author Contributions

IT-A wrote the initial draft which all authors critically revised and edited. All authors approved the final version.

## Funding

DF received funding from the Dutch Research Council (NWO) in the framework of the ENW PPP Fund for the top sectors (project Proteoform-resolved pharmacokinetics of biopharmaceuticals, no. 019.012).

## Conflict of Interest

IT-A is an employee of Genos Ltd, a private research organization that specializes in high-throughput glycomic analysis. ER is co-affiliated to Max Planck Institute for Dynamics of Complex Technical Systems and glyXera. He is founder and CEO of glyXera GmbH, providing high-performance glycoanalytical products and services.

The remaining authors declare that the research was conducted in the absence of any commercial or financial relationships that could be construed as a potential conflict of interest.

## Publisher’s Note

All claims expressed in this article are solely those of the authors and do not necessarily represent those of their affiliated organizations, or those of the publisher, the editors and the reviewers. Any product that may be evaluated in this article, or claim that may be made by its manufacturer, is not guaranteed or endorsed by the publisher.
